# Low thigh muscle strength in relation to myosteatosis in patients with type 2 diabetes mellitus

**DOI:** 10.1038/s41598-022-24002-1

**Published:** 2023-02-02

**Authors:** Yilong Huang, Jun Yan, Hongli Zhu, Zhenguang Zhang, Yuanming Jiang, Xuxiang Zhang, Yiran Wu, Jiahang Lu, Hanxue Cun, Bo He

**Affiliations:** 1 Department of Medical Imaging, The First Affiliated Hospital of Kunming Medical University, Kunming, China; 2 Department of Medical Imaging, The Affiliated Qujing Hospital of Kunming Medical University, Qujing, China; 3 Department of Endocrinology, The First Affiliated Hospital of Kunming Medical University, Kunming, China; 4 Department of Rehabilitation, The First Affiliated Hospital of Kunming Medical University, Kunming, China

**Keywords:** Biomarkers, Muscle, Type 2 diabetes

## Abstract

This study aimed to investigate the association of thigh muscle fat infiltration by quantitative MRI with muscle strength in patients with type 2 diabetes mellitus (T2DM). Seventy T2DM patients and sixty control subjects (71 males; age: 52 ± 8 years) underwent 3.0T MRI and isokinetic muscle strength measurements to obtain the skeletal muscle index (SMI), intermuscular adipose tissue (IMAT) proton density fat fraction (PDFF), intramuscular fat (IMF) PDFF, peak torque (PT) and total work (TW) of knee extensors and flexors. The differences of measurements between T2DM patients and asymptomatic volunteers were compared. Multivariate regression analysis was used to determine significant predictors of thigh extension and flexion strength. The SMI, IMAT and IMF PDFF of thigh muscles in T2DM patients were higher than that in the control group (*p* < 0.001), while PT and TW were lower than those in the control subjects (*p* < 0.05). Both IMF and IMAT PDFF were negatively correlated with PT, TW in participants with T2DM (extensors: *r* = − 0.72, − 0.70, *p* < 0.001;* r* = − 0.62, − 0.56, *p* < 0.05. flexors: *r* = − 0.37, − 0.43, *p* < 0.05; *r* = − 0.39, − 0.46, *p* < 0.05). Moderate and strong correlations between HOMA-IR and muscle strength measurements, muscle PDFFs were observed in extensors and flexors. IMF PDFF and age were the statistically significant predictor of PT and TW of extensors of thigh in multivariate regression analysis. Therefore, the thigh muscle PDFF increased was associated with muscle strength decreased in T2DM patients beyond SMI. Age are also important factors influencing thigh muscle PDFF and strength in T2DM patients.

## Introduction

Diabetes mellitus has become the third most common non‐communicable disease threatening human health globally, following only cardiovascular disease and cancer^1^. Type 2 diabetes mellitus (T2DM) is the most common type of diabetes, accounting for around 90% of all diabetes patients^2^. In general, excess circulating free fatty acids lead to ectopic fat deposition, such as the liver, skeletal muscle, heart, and pancreas, which results in insulin resistance (IR) in target organs^3^. Along with the progression of T2DM, lipid metabolism disturbance further exacerbates the occurrence of ectopic fat deposition^4^. Skeletal muscle is not only the largest organ and predilection site of ectopic fat deposition in T2DM, but also the main organ of energy metabolism^5^. When a large amount of ectopic fat is deposited in skeletal muscle, lipotoxicity can interfere with insulin signaling and impair mitochondrial function, which reduces glucose utilization by skeletal muscle, resulting in weakened muscle strength and decreased muscle mass, as well as induces IR and promotes the occurrence and development of T2DM^6^. Some studies showed that insulin receptor expression and function were downregulated and post‐receptor insulin signaling was defected in T2DM patients, affecting muscle and adipose tissue glucose uptake, glycogen synthesis and endogenous glucose production^7,8^. Besides, hyperglycemia, chronic inflammation, and oxidative stress in T2DM can also aggravate skeletal muscle fat infiltration^9^.

The thigh muscle is one of the largest and most important muscle groups in the human body, as it plays an important role in maintaining an upright posture, supporting body weight, and walking. T2DM patients can experience loss of muscle mass, strength, function and quality over time, particularly in the lower extremities^10^, which is associated with a deteriorating health status, reduced mobility, even sarcopenia and falls-related fractures^11,12^. Moreover, fat infiltration in the muscle may distort muscle architecture, further resulting in the loss of muscle strength^13^. In previous study, muscle strength was assessed using a relatively inexpensive handheld dynamometry or manual muscle test^14^. But these approaches present some limitations, such as strong subjectivity and low accuracy. Knee isokinetic muscle strength measurement represents the gold standard for assessing flexion and extension muscle strength of the lower extremity, as it is more efficient, repeatable and accurate^15^. However, the underlying connections between ectopic fat deposition in skeletal muscle, muscle strength, and IR are unclear.

Non-invasive and accurate quantitative assessment of ectopic fat deposition and muscle dysfunction of skeletal muscle in patients with T2DM is of great significance. Recently, magnetic resonance imaging (MRI) has been introduced as a preferred imaging modality for quantifying and localizing muscle fat^16^. The chemical shift encoding-based fat quantification techniques and MR spectroscopy (MRS) provide quantitative assessments of fat in several organs, which have even been considered “gold-standard” methods^17^. But disadvantages of in vivo MRS are its poor spectral resolution, long scanning time, and limited measurement range. Recently, advanced chemical shift-encoded MRI (CSE-MRI) has been reported to have the advantages of convenient measurement, short scanning time, accurate quantification, and good reproducibility, and can comprehensively evaluate fat content in the organs by proton density fat fraction (PDFF)^18^. Moreover, PDFF based on CSE-MRI technology has been confirmed to be in good agreement with MRS and histology^19^, and has been considered a reliable biomarker for non-invasive fat quantification^20^. Kromrey et al.^21^ found an inverse correlation between pancreatic steatosis quantified by PDFF and impaired pancreatic exocrine function. Given the above analysis, we hypothesized that quantitative MRI could precisely assess lower extremity muscle fatty infiltration in patients with T2DM and was associated with IR and the loss of lower extremity muscle strength.

Therefore, the purpose of this study was to evaluate the clinical value of quantitative MRI for assessing the level of ectopic fat deposition in T2DM thigh skeletal muscle and to explore the relationship between PDFF of thigh muscle, IR and reduced muscle strength. Furthermore, we compared the age- and sex-related difference in muscle atrophy, PDFF and isokinetic muscle strength of different thigh muscles in patients with T2DM.

## Materials and methods

### Participants and clinical data

From June 2020 to September 2021, 70 patients with T2DM and 60 healthy control subjects without T2DM were recruited for this prospective study (71 males, 59 females; mean age: 52.02 ± 8.73 years; age range: 35–69 years). The control cohort was matched based on age, sex and body mass index (BMI). This study was approved by the ethics committee of the first affiliated hospital of Kunming Medical University (Approval Number: 2018‑L-86). All methods were performed in accordance with relevant guidelines and regulations. All enrolled T2DM patients and volunteers signed the informed consent form. All participants underwent thigh MRI examinations and isokinetic muscle strength measurement of the left lower limb. Physical activity was measured using the Short-form International Physical Activity Questionnaire. All participants have the dominant right foot. The inclusion criteria of T2DM patients were as follows: (1) Diagnosis of T2DM followed the American Diabetes Association criteria. (2) Moderate-intensity activity, 600–1500 MET-min/week. The inclusion criteria of healthy control subjects without T2DM were as follows: (1) No history of T2DM, no T2DM-related clinical manifestations and normal fasting blood glucose. (2) Moderate-intensity activity. The exclusion criteria of all participants were contraindications for MRI; surgery, trauma, fracture, tumor, infection deformity and other musculoskeletal diseases of lower extremity; Pregnancy; Binge drinker (alcohol ≥ 30 g/d); Long-term use of hormones; Type 1 diabetes mellitus and other endocrine disorders. According to the median age of the included participants, participants under 51 years old are classified as the young group, and 51 years old or older are the old group. Age, sex, height, weight, BMI, homeostasis model assessment of insulin resistance (HOMA-IR) index, comorbid conditions and medications were recorded for all participants. HOMA-IR was calculated using the formula HOMA-IR = (fasting blood glucose × fasting insulin)/22.5. All participants fasted and discontinued all diabetes medications overnight for more than 12 h before blood collection. Table 1 shows the baseline clinical characteristics of the participants.

**Table 1:** Comparison of clinical characteristics between control volunteers without T2DM and T2DM patients ($\overline{x }\hspace{0.17em}$± s).

Characteristics	Control volunteers (*n* = 60)	T2DM patients (*n* = 70)	*p*
Male/female	30/30	40/30	0.304
Age (year)	50.72 ± 8.73	52.71 ± 7.37	0.117
BMI (kg/m^2^)	26.07 ± 2.30	27.23 ± 2.71	0.754
Male	25.75 ± 2.32	27.13 ± 2.47	0.471^#^
Female	26.40 ± 2.25	27.68 ± 3.24	
Young	25.73 ± 2.02	27.07 ± 2.62	0.393^#^
Old	26.47 ± 2.56	27.65 ± 2.99	
HOMA-IR	NA	7.30 ± 6.23	NA

*T2DM* type 2 diabetes mellitus, *BMI* body mass index, *HOMA-IR* homeostasis model assessment of insulin resistance.

^#^Intragroup comparison in T2DM patients.

### MR data acquisition

Muscle MRI of the left thigh was conducted within 48 h after blood sample collection. All MRI experiments were performed using a 3.0T MR system (Discovery 750w, GE Healthcare, USA). A 24-channel phased array body coil was used for T2DM patients and healthy control volunteers. Within the MRI, participants were placed in the supine position and bilateral lower limbs are completely in a relaxed state. MRI scanning for participants included axial T1-weighted imaging (T1WI), coronel T2-weighted imaging (T2WI) and axial iterative decomposition of water and fat with echo asymmetry and least-squares estimation (IDEAL-IQ) of the middle of thigh muscles. The MRI protocols of participants are summarized in Table 2.

**Table 2:** MRI scan parameters.

Images	TR (ms)	TE (ms)	ST (mm)	SL (mm)	BW(Hz)	FA (°)	Matrix	NEX
Axial T1WI	740.00	12.89	3.0	4.0	162.77	111	512 × 512	1.0
Coronal T2WI	2994.00	69.82	6.0	7.5	325.51	111	512 × 512	6.0
Axial IDEAL-IQ	9.80	1.3–7.2	4.0	4.0	868.05	4	256 × 256	0.5

*T1WI* T1-weighted imaging, *T2WI* T2-weighted imaging, *TR* time of repetition, *TE* echo time, *ST* slice thickness, *SL* slice increment, *BW* band width, *FA* flip angle, *NEX* number of excitations.

### Image analyses

**Figure 1:**
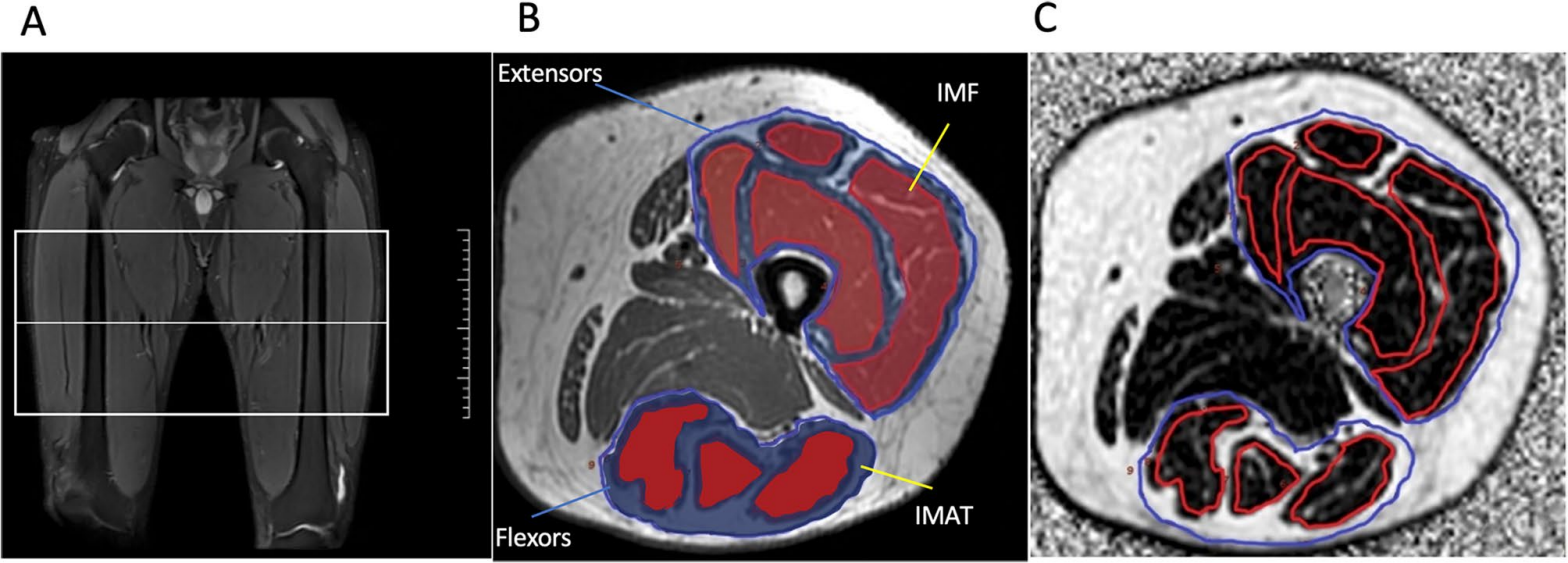
Magnetic resonance image scanning and muscles MRI ROI delineation. (**A**) MRI scanning range of the thigh (white box). (**B**) Axial T1WI showing segmentation of thigh muscle and fat. IMF, intramuscular fat; IMAT, intermuscular adipose tissue. (**C**) PDFF maps of left thigh muscle.

All raw MR images were processed on a commercially available workstation (Advantage Windows 4.6, GE Medical Systems, USA). Muscle cross-sectional area (CSA) and PDFF values of left extensors (quadriceps) and flexors (hamstrings) muscles were obtained on a region of interest (ROI) in one single slice of the thigh central level (Fig. 1A). After the PDFF maps were matched with T1WI, the muscle ROIs of the extensors and flexors were delineated along the edge of the myofascial on the T1WI. Because T1WI showed myofascial more clearly. The muscle CSA was measured on the axial T2WI map, and the same ROI was copied to the fat fraction map to obtain the PDFF values of intermuscular adipose tissue (IMAT) and intramuscular fat (IMF). ROI definition of IMAT and IMF refers to Karampinos et al.^22^. Further, the ROI of each muscle in the extensors and flexors was drawn separately, and the IMF PDFF value of each muscle was obtained (Fig. 1B,C). The color bar of PDFF was used with default setting from 0 to 100%. The higher the gray level, the higher PDFF percent. The CSA was normalized by height to obtain the Skeletal Muscle Index (SMI) [SMI = CSA/height^2^ (cm^2^/m^2^)]. All of these measurements were performed by two blinded, independent, and experienced radiologists. The two radiologists manually delineated the shape of the left knee extensors and knee flexors. The average of the two measurements was calculated and used for later analysis.

### Isokinetic muscle strength

Muscle strength was measured using an isokinetic dynamometer (HUMAC NORM Testing & Rehabilitation System, Computer Sports Medicine Inc., U.S.A.). All participants were permitted to perform one repetitions for familiarization prior to formal testing. Participants maintained an upright sitting position during the test while the knee joint was aligned with the mechanical axis of the dynamometer. The waist is close to the seat back and the angle between the seat and the dynamometer is kept at 90°. Straps were used to stabilize the bilateral lower limbs limb and waist. Two tests were performed on isokinetic knee extensions and flexions at two constant angular speeds of 60°/s and 180°/s. The first test was repeated 5 times at 60°/s. The second test was repeated 15 times at 180°/s. To avoid fatigue, the rests interval was 10 s between repetitions. Participants were encouraged to make their maximum efforts for each flexion and extension. The peak torque (PT) and total work (TW) of the left knee were analyzed and calculated by the software^23,24^. PT was the maximum of five repetitions in the first test. TW was determined as the total work accomplished for 15 repetitions in the second test.

### Statistical analysis

Statistical analyses were performed with SPSS 26.0. The metering data are expressed by Mean ± SD. Comparisons between patients with T2DM and healthy volunteers were determined using the independent-sample t-test. Pearsons correlations and Spearman’s rank correlations were computed between age, sex, BMI, SMI, muscles PDFF, HOMA-IR, PT and TW. One-way analysis of variance (ANOVA) or Kruskal–Wallis H test was employed for the comparisons among multiple groups, and Bonferroni test was utilized for the post hoc test after ANOVA. Multivariate analysis was performed using a multiple linear regression analysis. A *p*-value < 0.05 was reported statistically significant.

## Results

### Baseline characteristics and quantitative MRI measurements

**Figure 2:**
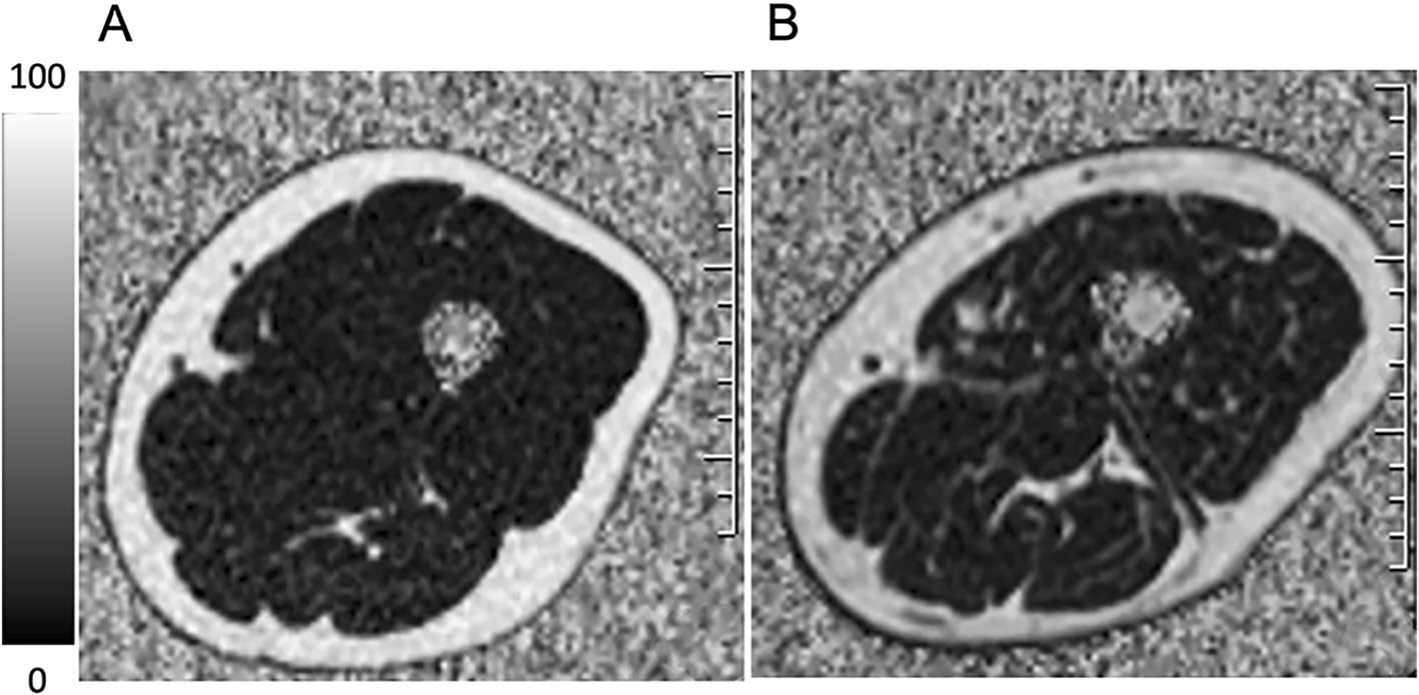
Representative MRI PDFF of thigh in control and T2DM. (**A**) Control volunteer without T2DM, Female, 55 years old. IMAT PDFF_extensors_ = 10.60%, IMF PDFF_extensors_ = 2.75%, IMAT PDFF_flexors_ = 17.00%, IMF PDFF_flexors_ = 4.10%. (**B**) T2DM patient, Female, 55 years old. IMAT PDFF_extensors_ = 18.90%, IMF PDFF_extensors_ = 6.05%, IMAT PDFF_flexors_ = 23.08%, IMF PDFF_flexors_ = 10.57%. White represents high PDFF values.

There were no differences in sex, age and BMI between control volunteers without T2DM and T2DM patients (Table 1). There was no significant difference in BMI between healthy control volunteers and T2DM patients in different genders and ages (*p* > 0.05, Table 1). The SMI, IMAT PDFF and IMF PDFF of the thigh extensors and flexors of T2DM were higher than those of healthy control volunteers (*p* < 0.01, Table 3). The PDFF maps showed that the thigh extensors and flexors PDFFs were increased in patients with T2DM (Fig. 2). The thigh extensors and flexors of T2DM patients had reduced PT and TW compared with normal controls (*p* < 0.05, Table 3).

**Table 3:** Differences in MRI parameters and isokinetic muscle strength measurements of thigh muscles between volunteers without T2DM and T2DM patients ($\overline{x }\hspace{0.17em}$± s).

Parameters	Muscles	Control volunteers	T2DM patients	*p*
SMI (cm^2^/m^2^)	Extensors	21.17 ± 3.42	23.60 ± 3.63	< 0.001***
	Flexors	9.31 ± 1.45	10.47 ± 1.70	< 0.001***
IMAT PDFF (%)	Extensors	11.06 ± 3.31	13.03 ± 3.74	0.002**
	Flexors	19.04 ± 5.63	23.42 ± 4.46	< 0.001***
IMF PDFF (%)	Extensors	3.20 ± 0.98	4.08 ± 1.44	< 0.001***
	Flexors	5.39 ± 1.90	7.68 ± 2.30	< 0.001***
PT (N·m)	Extensors	59.17 ± 15.70	42.67 ± 15.71	< 0.001***
	Flexors	33.20 ± 10.98	23.47 ± 7.35	0.008**
TW (J)	Extensors	335.21 ± 129.82	256.00 ± 166.46	0.047*
	Flexors	294.33 ± 158.40	181.13 ± 91.24	0.025*

*SMI* skeletal muscle index, *PDFF* proton density fat fraction, *PT* peak torque, *TW* total work.

*p < 0.05; **p < 0.01; ***p < 0.001.

### Difference in SMI, PDFF, PT and TW regarding sex and age

**Figure 3:**
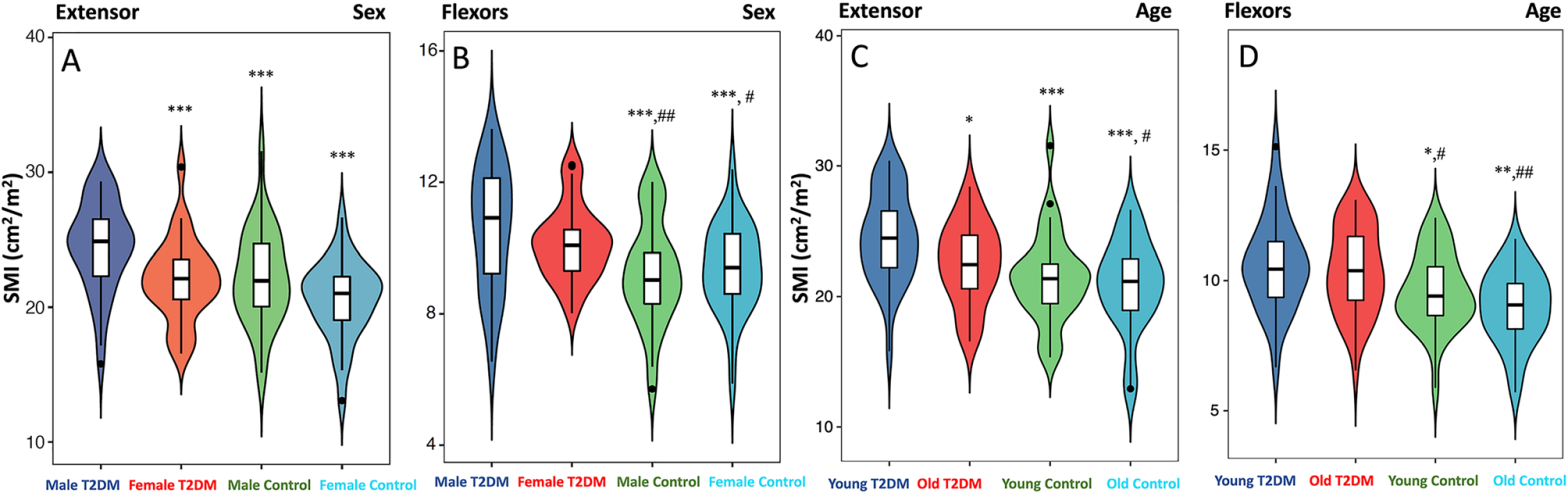
Difference in SMI regarding sex and age. (**A**) Male and female extensors SMI in T2DM patients and control group; (**B**) Male and female flexors SMI in T2DM patients and control group; (**C**) Young and old extensors SMI in T2DM patients and control group; (**D**) Young and old flexors SMI in T2DM patients and control group; *p < 0.05, **p < 0.01, ***p < 0.001, *vs.* Male/Young T2DM patients. #*p* < 0.05, ##*p* < 0.01, *vs.* Female/Old T2DM patients.

**Figure 4:**
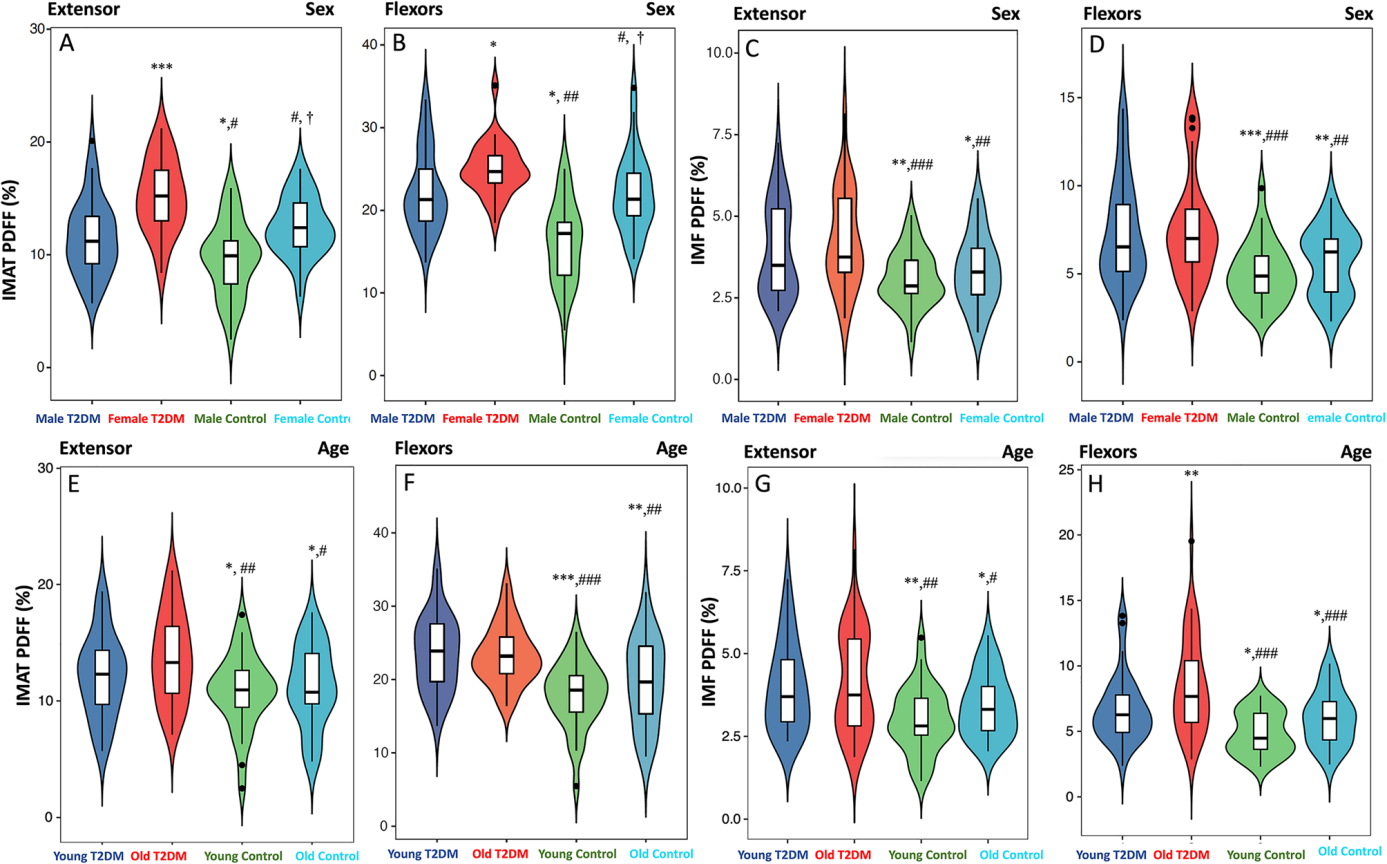
Difference in PDFF of thigh muscle regarding sex and age. (**A**,**B**) Male and female muscles IMAT PDFF in T2DM patients and control group; (**C**,**D**) Male and female muscles IMT PDFF in T2DM patients and control group; (**E**,**F**) Young and old muscles IMAT PDFF in T2DM patients and control group; (**G**,**H**) Young and old muscles IMT PDFF in T2DM patients and control group; *p < 0.05, **p < 0.01, ***p < 0.001, *vs.* Male/Young T2DM patients. #*p* < 0.05, ##*p* < 0.01, ###*p* < 0.001, *vs.* Female/Old T2DM patients. †*p* < 0.05, *vs.* Male/Young control group.

**Figure 5:**
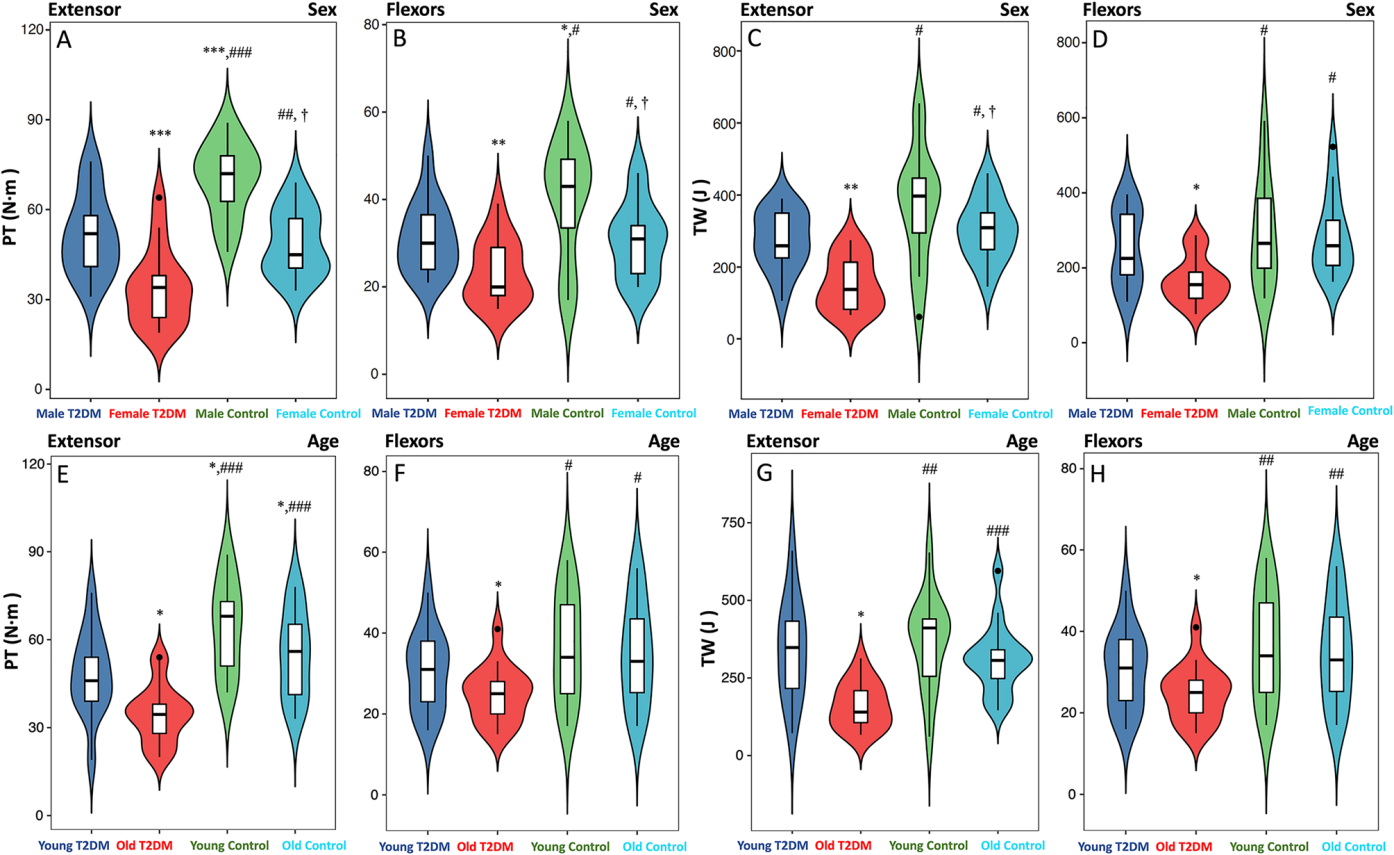
Difference in isokinetic muscle strength regarding sex and age. (**A**,**B**) Male and female muscles PT in T2DM patients and control group; (**C**,**D**) Male and female muscles TW in T2DM patients and control group; (**E**,**F**) Young and old muscles PT in T2DM patients and control group; (**G**,**H**) Young and old muscles TW in T2DM patients and control group; *p < 0.05, **p < 0.01, ***p < 0.001, *vs.* Male/Young T2DM patients. #*p* < 0.05, ##*p* < 0.01, ###*p* < 0.001, *vs.* Female/Old T2DM patients. †*p* < 0.05, *vs.* Male/Young control group.

In the healthy control group, there was no significant difference in the SMI of extensors and flexors in different genders and ages (Fig. 3, *p* > 0.05). But female and old T2DM patients have a lower extensors SMI than male and young T2DM patients (Fig. 3, *p* < 0.05). In addition, the healthy male had lower IMAT PDFF of the extensors and flexors than healthy female (Fig. 4A,B, *p* < 0.05). But there was no sex difference in the IMF PDFF (Fig. 4C,D, *p* > 0.05). The extensors and flexors PDFF of the old were slightly higher than that of the young in both controls and T2DM patients, but the difference was not statistically significant (Fig. 4E–H, all *p* > 0.05). Compared with the healthy controls, T2DM patients differing in sex and age had higher IMAT and IMF PDFF of the extensors and flexors, and the female T2DM patients had higher IMAT PDFF than males (Fig. 4, *p* < 0.05). In the healthy controls and the T2DM patients, the PT and TW of the male extensors and flexors were higher than those of the female (Fig. 5A–D, *p* < 0.05). Both PT and TW in extensor and flexor muscles were decreased significantly in male and female T2DM patients compared to healthy controls (Fig. 5A–D, *p* < 0.05). Regardless of young or old, the PT and TW of extensor and flexor muscles showed a significant decrease in the T2DM patients. And old T2DM patients were lower than the young group in the PT and TW of muscles (Fig. 5E–H, *p* < 0.05).

### Correlations of sex, age, BMI, HOMA-IR, SMI, muscle PDFF, PT and TW

**Figure 6:**
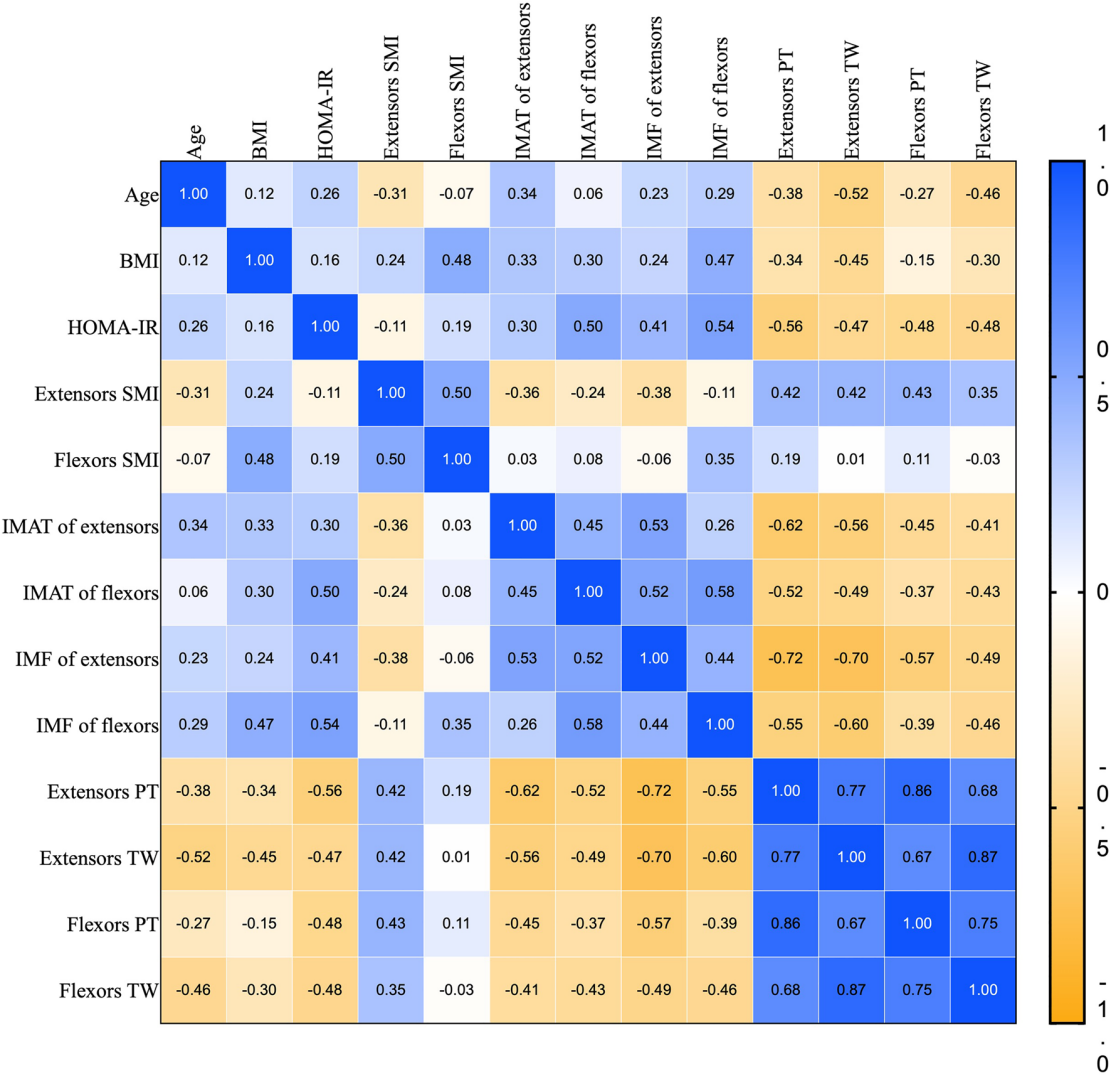
Heatmaps of correlation coefficients clinical characteristics, muscle PDFF and isokinetic muscle strength in patients with T2DM. BMI, body mass index; HOMA-IR, homeostasis model assessment-insulin resistance; SMI, skeletal muscle index. IMAT, intermuscular adipose tissue; IMF, intramuscular fat; PT, peak torque; TW, total work.

HOMA-IR was also moderately and strongly positively correlated with IMAT and IMF PDFF (*r* values ranged from 0.30 to 0.54, *p* < 0.05). PT and TW were negatively correlated with sex, age and HOMA-IR. IMAT and IMF PDFF of extensors muscle were strongly negatively correlated with extensors PT and TW in extensors (*r* values ranged from − 0.52 to − 0.72, *p* < 0.05), and the correlation between IMF PDFF and PT and TW was higher than that of IMAT PDFF. IMF and IMAT PDFF of flexors were moderately negatively correlated with PT and TW in flexors (*r* values ranged from − 0.37 to − 0.46, *p* < 0.05), and the correlation between IMF PDFF of flexors and PT and TW was slightly higher than that of IMAT PDFF. The extensors were more correlated with lower PDFF and PT and TW than the flexors. Age was weakly and moderately positively correlated with IMF PDFF and extensors IMAT (*r* values ranged from 0.23 to 0.34, *p* < 0.05). Figure 6 shows the relationships between age, BMI, HOMA-IR, SMI, muscle PDFF, PT and TW.

### Multiple linear regression analysis

The multiple linear regression analysis is presented in Table 4. Age and IMF PDFFs were independent factors of extensors PT and TW in T2DM patients (*p* < 0.05). But age, sex, BMI, HOMA-IR, SMI, IMAT and IMF PDFF were not independent factors of flexors PT and TW in T2DM patients (*p* > 0.05).

**Table 4:** Multiple linear regression analysis of PT and TW of thigh muscles in T2DM patients.

Parameters	PT				TW			
	R^2^_adj_	Standardized β	95%CI	*p*	R^2^_adj_	Standardized β	95%CI	*p*
**Extensors**								
Age	0.491	− 0.117	− 0.51, − 1.07	0.047*	0.457	− 0.167	− 4.34, − 2.94	0.034*
Sex		− 0.083	− 20.37, 9.06	0.434		− 0.080	− 171.42, 144.03	0.859
BMI		0.016	− 1.43, 1.58	0.918		0.123	− 10.86, 22.58	0.451
HOMA-IR		− 0.087	− 1.92, 1.11	− 0.554		− 0.094	− 21.28, 11.92	0.566
SMI		0.159	− 0.87, 2.45	0.336		0.209	− 7.16, 29.20	0.222
IMAT PDFF		− 0.247	− 2.59, 0.48	0.169		− 0.158	− 23.91, 9.65	0.388
IMF PDFF		− 0.509	− 8.97, − 1.30	0.011*		− 0.542	− 99.90, − 15.99	0.009*
**Flexors**								
Age	0.019	− 0.029	− 0.72, 0.82	0.502	0.116	− 0.310	− 3.09, 16.43	0.171
Sex		− 0.373	− 18.27, 2.99	0.150		− 0.329	− 227.06, 46.67	0.186
BMI		0.130	− 0.95, 1.76	0.542		− 0.031	− 18.46, 15.91	0.879
HOMA-IR		− 0.074	− 1.63, 1.17	0.738		− 0.101	− 21.96, 13.58	0.630
SMI		0.259	− 0.94, 3.96	0.214		0.067	− 25.83, 36.32	0.730
IMAT PDFF		− 0.143	− 1.61, 1.01	0.642		− 0.067	− 18.52, 14.77	0.818
IMF PDFF		− 0.366	− 3.29, 0.86	0.238		− 0.323	− 43.15, 9.48	0.199

*BMI* body mass index, *SMI* skeletal muscle index, *HOMA-IR* homeostasis model assessment-insulin resistance, *PDFF* proton density fat fraction, *PT* peak torque, *TW* total work.

*p < 0.05.

## Discussion

Based on quantitative MRI, our study found that T2DM patients had increased fat content in the thigh muscles and decreased isokinetic muscle strength. The decrease of thigh muscle strength in T2DM patients was positively correlated with muscle ectopic fat deposition and IR. Furthermore, age in T2DM patients were associated with muscle ectopic fat deposition and decreased muscle strength.

Compared with normal volunteers without T2DM, the IMAT and IMF PDFF of thigh extensors and flexors in T2DM patients were significantly increased, and the results of increased IMAT in the lower extremity muscles of T2DM patients were consistent with the previous study^5^. Kiefer et al.^25^ found increased PDFF of abdominal muscles in both T2DM and prediabetic subjects compared to healthy subjects. Our results confirmed that ectopic fat deposition occurred in the thigh skeletal muscle of T2DM patients and could be accurately quantified by PDFF. It is often assumed that reduced muscle mass is common in T2DM patients^26^, the submyofascial SMI of the thigh muscles in T2DM patients in this study was slightly higher than in controls. But this inconsistent situation is not representative of the thigh muscle tissue of T2DM patients has increased. The SMI of thigh muscles may be attributed to the increase in IMF and IMAT, which may mask muscle tissue atrophy^27^. Besides, the increasing percentages of IMF (36.90%) is higher than IMAT (18.96%) in T2DM patients compared to healthy control subjects. The study by Karampinos and Bittle et al. also reported similar results^5,22^. Compared with age- and sex-matched non-T2DM subjects, T2DM patients had significantly decreased maximal strength and endurance of both thigh flexors and extensors, and the extensors changes were more prominent. Decreased muscle strength in patients with T2DM may be associated with insulin resistance, muscle fat deposition, mitochondrial dysfunction, muscle loss, and neuromuscular impairment^28,29^. Decreased activity in T2DM patients mainly results in decreased use of anti-gravitational muscles, especially the thigh extensors^28,30^, so the decrease in extensors muscle strength is more significant in T2DM patients, which may further promote the increase in muscle ectopic fat deposits.

Recently, much attention has been paid to the causes and mechanisms of decreased skeletal muscle strength in T2DM. Previous studies have considered muscle mass as a decisive factor in muscle strength^31^, but decreased muscle strength is also associated with neuromuscular dysfunction and increased muscle fat^32^. Askary-Ashtian et al. suggested the decreased muscle strength in T2DM patients was the result of decreased muscle mass^29^. From our results, the IMF PDFF may be a better predictor of changes in muscle strength than muscle mass in T2DM patients. In this study, we further analyzed the correlation between T2DM thigh muscle and isokinetic muscle strength. The results showed that the PT and TW of flexors and extensors in T2DM patients were negatively correlated with IMAT and IMF, and the correlation between IMF PDFF was more significant. Meanwhile, multiple linear regression analysis showed that only the IMF PDFF of the extensor group was still associated with PT and TW. This indicates that compared with IMAT, IMF can better reflect the changes of maximal muscle strength and endurance of thigh muscles in T2DM patients. Regarding the mechanism of muscle dysfunction caused by increased IMF fat, it is currently believed to be related to chronic low-level inflammation and inhibition of central activation. IMF adipose tissue accumulation produces inflammatory mediators (IL-6, THF-α, etc.) that cause low-level inflammation in muscle and inhibit central activation^33^, reducing the responsiveness of myofilaments to calcium, leading to muscle dysfunction^34^.

In this study, we account for the potential effects of sex and age on muscle fat in T2DM patients. The results may indicate that ectopic fat deposition in skeletal muscle and decreased muscle strength in T2DM are gender-neutral. In previous studies, Jeon and Chen et al. investigated risk factors for decreased lower extremity muscle strength, grip strength, and back strength in patients with T2DM and found that sex differences were not associated with decreased skeletal muscle strength^35,36^. However, unlike sex, age may be a risk factor for ectopic fat deposition in skeletal muscle and decreased muscle strength in patients with T2DM. After reaching a peak in early adulthood (35–40 years of age), muscle strength gradually declines^31^. Meanwhile, aging humans also experience changes in muscle composition, leading to increased muscle fat content^13,37^. Van et al. have shown that T2DM accelerates age-related impairment of muscle function and loss of muscle mass^38^. In this study, both younger and elderly T2DM patients had increased skeletal muscle PDFF compared with healthy controls, but the isokinetic muscle strength of the thigh extensors and flexors muscle in the elderly T2DM patients was decreased more significant than that in the young. The older the age was, the lower the muscle strength in patients with T2DM.

The present study has a few limitations. Firstly, some of the patients with T2DM recruited in this study were treated with medicines, which might have had an effect on the results. The effect of internal medicine treatment on muscle fatty infiltration and strength in T2DM patients is of great interest. The healthy control subjects in this study are not T2DM patients, who did not test fasting insulin. An underlying IR may also happen in a small part of control subjects. Furthermore, this study is a cross-sectional analysis, making it difficult to establish a cause-effect relationship between thigh muscle ectopic fat deposition and muscle strength and IR. In addition, we measured muscle dimensions and PDFF of most muscles of the thigh at the extensors and flexors only, in order to match the results of MRI quantification and isokinetic muscle strength measurements of the extensors and flexors. However, this does not mean that the remaining muscles were not altered in T2DM patients.

Using quantitative MRI, increased IMAT and IMF in thigh muscles are important consideration for the IR and decreased isokinetic muscle strength in patients with T2DM, and the effects were more significant in the older T2DM patients. This study may provide new strategies for the early implementation of individualized exercise training interventions in patients with T2DM. The thigh muscles PDFF should also be evaluated as a treatment outcome to avoid the aggravation of IR and loss of muscle mass and strength reducing the risk of falls and fractures.

## Data Availability

The data supporting this article are available from the corresponding author on reasonable request.

## Abbreviations

BMI: Body mass index
CSA: Cross-sectional area
CSE-MRI: Chemical shift-encoded MRI
FBG: Fasting blood glucose
FINS: Fasting insulin
HOMA-IR: Homeostasis model assessment of insulin resistance
IDEAL-IQ: Iterative decomposition of water and fat with echo asymmetry and least-squares estimation
IMAT: Intermuscular adipose tissue
IMF: Intramuscular fat
IR: Insulin resistance
MRI: Magnetic resonance imaging
MRS: Magnetic resonance spectroscopy
PDFF: Proton density fat fraction
PT: Peak torque
ROI: Region of interest
SMI: Skeletal muscle index
T1WI: T1-weighted imaging
T2DM: Type 2 diabetes mellitus
T2WI: T2-weighted imaging
TW: Total work
